# Recent Advances in Epigenetics of Age-Related Kidney Diseases

**DOI:** 10.3390/genes13050796

**Published:** 2022-04-29

**Authors:** Feng Liu, Jiefang Chen, Zhenqiong Li, Xianfang Meng

**Affiliations:** 1 Department of Nephrology, Union Hospital, Tongji Medical College, Huazhong University of Science and Technology, Wuhan 430022, China; liufenguro@163.com; 2 Department of Neurology, Union Hospital, Tongji Medical College, Huazhong University of Science and Technology, Wuhan 430022, China; chenjiefangneuro@163.com; 3 Department of Neurobiology, Institute of Brain Research, School of Basic Medical Sciences, Tongji Medical College, Huazhong University of Science and Technology, Wuhan 430030, China

**Keywords:** epigenetics, histone modification, DNA methylation, non-coding RNA regulation, age-related kidney diseases

## Abstract

Renal aging has attracted increasing attention in today’s aging society, as elderly people with advanced age are more susceptible to various kidney disorders such as acute kidney injury (AKI) and chronic kidney disease (CKD). There is no clear-cut universal mechanism for identifying age-related kidney diseases, and therefore, they pose a considerable medical and public health challenge. Epigenetics refers to the study of heritable modifications in the regulation of gene expression that do not require changes in the underlying genomic DNA sequence. A variety of epigenetic modifiers such as histone deacetylases (HDAC) inhibitors and DNA methyltransferase (DNMT) inhibitors have been proposed as potential biomarkers and therapeutic targets in numerous fields including cardiovascular diseases, immune system disease, nervous system diseases, and neoplasms. Accumulating evidence in recent years indicates that epigenetic modifications have been implicated in renal aging. However, no previous systematic review has been performed to systematically generalize the relationship between epigenetics and age-related kidney diseases. In this review, we aim to summarize the recent advances in epigenetic mechanisms of age-related kidney diseases as well as discuss the application of epigenetic modifiers as potential biomarkers and therapeutic targets in the field of age-related kidney diseases. In summary, the main types of epigenetic processes including DNA methylation, histone modifications, non-coding RNA (ncRNA) modulation have all been implicated in the progression of age-related kidney diseases, and therapeutic targeting of these processes will yield novel therapeutic strategies for the prevention and/or treatment of age-related kidney diseases.

## 1. Introduction

With the rapid development of social economy and the improvement in health care, the life expectancy of the general population has continued to increase, resulting in a significantly increasing proportion of the elderly population [1]. According to World Health Organization (WHO) statistics, it is estimated that there will be approximately two billion people over 60 years old worldwide by 2050 [2,3,4]. Among all the issues caused by aging, renal aging is becoming nonnegligible. Although the aging process does not directly cause kidney disease, aging kidneys are more susceptible to various adverse factors such as high blood pressure, diabetes, obesity, or primary renal disorders, which may contribute to the development of renal pathologies. Aging is associated with an inevitable time-dependent decline in kidney function [5,6]. The main pathological characteristics of the aged kidney include arteriosclerosis, glomerulosclerosis, tubular atrophy, and interstitial fibrosis [7]. Specifically, numerous studies have indicated that aging is recognized as a major contributor to the increased incidence of acute kidney injury (AKI) and chronic kidney disease (CKD) in the overall population [7,8,9]. Furthermore, the incidence of deaths due to renal dysfunction is increasing globally in parallel with the aging population. However, the underlying mechanisms of renal aging have not been clearly elucidated yet, and remain the focus of current research. Thus, wisely addressing the challenges of population aging requires identifying the root causes of this phenomenon as well as effectively solving the issues, which will bring huge social benefits.

So far, several mechanisms have been found to be closely associated with age-associated organ function decline including genome instability, telomere attrition, epigenetic alterations, loss of proteostasis, dysregulated nutrient sensing, mitochondrial dysfunction, cellular senescence, and stem cell exhaustion as well as altered intercellular communication [6,10], among which epigenetic changes are the newly identified hallmarks of aging in the past decade [6,11]. Epigenetics mainly refers to the study of molecular processes that regulate gene expression as well as phenotype without changing the primary DNA sequence, which mainly involves DNA methylation, histone post-translational modifications, chromatin remodeling, and regulation by non-coding RNAs (ncRNAs). In recent years, the critical role of epigenetic alterations has received increasing attention for its involvement in various disease processes as well as normal physiological functions. Moreover, it is now generally accepted that DNA methylation and histone modification are imprinted during embryonic development and must be maintained in adults for health and phenotypic stability [12]. Meanwhile, growing evidence suggests that cellular and organismal aging is closely linked to profound changes in a series of epigenetic modifications, leading to altered gene expression patterns [13,14]. In particular, accumulating evidence has demonstrated that epigenetic alterations such as DNA methylation [15,16,17,18], histone modifications [19,20], and regulation of ncRNAs [21,22,23] are involved in the initiation and development of renal aging processes [24]. Recently, aberrant epigenetic modifications, mainly including DNA methylation [11,17], histone modifications [20,25,26], and regulation of ncRNAs [27,28] have also been increasingly implicated in driving age-related kidney disorders. However, no previous systematic review has been performed to systematically generalize the relationship between epigenetics and age-related kidney diseases.

Epigenetics is a rapidly growing field of research and there is a profound interest in exploiting the epigenetics phenomena as diagnostic biomarkers or therapeutic targets of aging-related diseases [29]. In this review, we summarize the current understanding of the pathophysiological roles of epigenetics, particularly focusing on DNA methylation, histone modifications, and ncRNAs modulation, in age-related kidney diseases. At the same time, we focus on the promising use of these epigenetic alterations as potential tools for the early diagnosis, treatment, and prevention of age-related kidney diseases. Taken together, we suggest that epigenetics plays a key role in the aging process and could be used as potential therapeutic targets for the treatment of age-related kidney disease, which warrants further investigation to advance relevant knowledge.

## 2. The Phenotype and Mechanism of Renal Aging

### 2.1. Molecular Mechanisms of Aging

Aging is associated with an inevitable time-dependent decline in cellular, tissue, and organ functions [30], which has attracted the wide attention and curiosity of researchers throughout the entire history of humankind [6]. Although the free radical theory of aging was proposed as early as in 1956 by Denham Harman [31], it was not until 1983, when the first long-lived strains were isolated from Caenorhabditis elegans, that a new era in aging research began [32]. Currently, the proposed mechanisms that contribute to the aging process include genomic instability, telomere attrition, epigenetic alterations, loss of proteostasis, deregulated nutrient sensing, mitochondrial dysfunction, cellular senescence, and stem cell exhaustion as well as altered intercellular communication. In addition, the aging process is also profoundly influenced by external factors [33] such as bad living habits and adverse environmental factors, which are also associated with the aging of the body [34,35]. In contrast, healthy lifestyle habits such as good eating habits and moderate exercise can effectively delay aging and improve the quality of life of aging populations [34,35]. In spite of the numerous theories that have been proposed during the past four decades to explain the phenomenon of the aging process, the exact mechanisms that drive the fundamental process of aging are not fully understood yet [36,37].

Although the human aging process is associated with a gradual decline in the function of a variety of organ systems, substantial variation exists among people with the same age group due to a combination of genetic and epigenetic factors as well as environmental factors [24]. As such, there is no gold standard for determining what constitutes healthy aging, nor can a single biomarker provide a valid and reliable measure of biological aging [38]. Over the past several decades, a series of high potential candidate biomarkers of aging have been proposed and evaluated, but none of them have turned out to be universally applicable [39,40,41]. Clearly, more in-depth research is needed to obtain a systemic understanding of the underlying mechanisms of the aging process to provide an accurate prediction of aging and identify individuals at high risk of developing age-associated diseases or disabilities.

### 2.2. Pathological Characteristic of Aging Kidney

**Figure 1:**
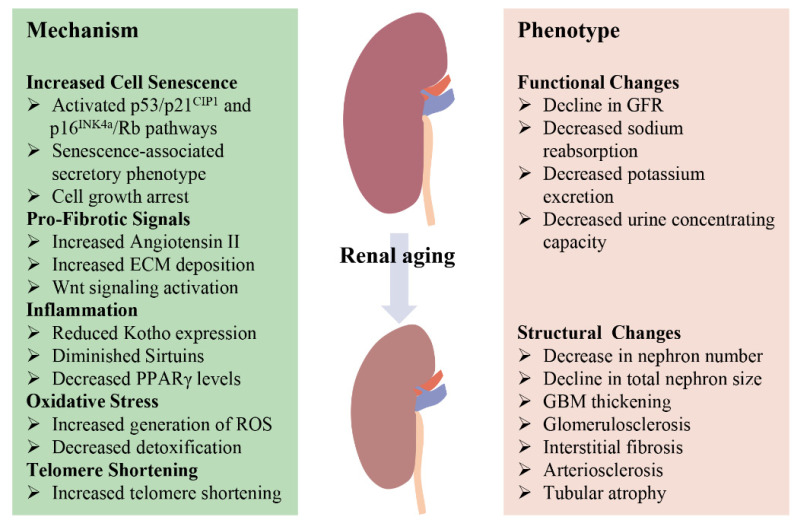
Schematic diagram of the main mechanisms and phenotype of renal aging. With increasing age, there are significant changes in both the function and structure of the kidney. Multiple age-related pathways contribute to altered renal outcomes in the elderly.

Aging is associated with an inevitable time-dependent decline in renal function and increased susceptibility to various acute or chronic kidney diseases [5,6], imposing a huge burden on health care systems globally. In order to discover efficient methods to delay renal aging, it is becoming very necessary to identify the factors driving age-associated changes in the kidney [3,42]. The health of the kidney depends on various factors including genetic background, gender, race, oxidative stress, and chronic inflammation as well as epigenetic factors (e.g., DNA methylation, histone modifications), all of which play a key role in the kidney aging process [43]. Although data generated from both cell and animal experiments indicate multiple pathways of potential importance for renal aging (Figure 1), data supporting their involvement in humans are currently rare, with additional research warranted.

With increasing age, there are irreversible alterations in the kidney at both microscopic and macroscopic levels as well as clinical and functional. It has been reported that renal weight decreases from the third to eighth decade of life, with the steepest decline occurring after the age of 50 [44,45], but imaging analysis indicates that the kidney parenchymal volume remains unchanged [46]. This may be, in large, attributed to a compensatory hypertrophy of unaffected nephrons in response to the loss of nephrons induced by glomerulosclerosis and tubular atrophy [47]. A number of structural changes occur in the kidney with aging, as demonstrated in Figure 1, mainly including a reduced nephron number, decreased total nephron size, glomerular basement membrane (GBM) thickness, glomerulosclerosis, interstitial fibrosis, arteriosclerosis, and tubular atrophy, which ultimately leads to decreased renal plasma flow (RPF) and glomerular filtration rate (GFR) [3,48]. Moreover, aging kidneys exhibit increased vulnerability to adverse conditions such as oxidative stress, inflammatory mediators, and fibrotic factors [49,50].

Collectively, these age-related structural and functional changes in the kidney may inevitably predispose the kidneys to various acute or chronic kidney disorders.

### 2.3. Age-Related Kidney Dysfunction

**Figure 2:**
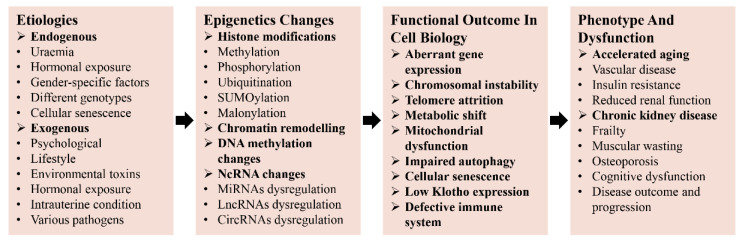
The epigenetic landscape mediates the interplay between etiologies and age-related renal dysfunctions. Both exogenous and endogenous etiologies can lead to changes in the epigenetic landscape that impact renal aging and physiology.

Toward a better understanding of the aging process to effectively intervene in it, distinguishing the difference between the natural aging process and age-related dysfunction is especially important [51]. This point is highly pertinent to a variety of age-related diseases, but is especially relevant in the context of kidney disease. Although aging itself does not cause kidney disease, age-related structural and functional changes may predispose aging individuals to various kidney diseases [7]. For example, under adverse conditions such as ischemia-reperfusion injury (IRI) and nephrotoxicity insult toxins, elderly people have increased susceptibility to AKI [42,52]. Several studies have demonstrated that the incidence of CKD in the elderly is 3–13 times higher than that in younger individuals [53,54]. Besides, there is evidence that cellular senescence is engaged in the pathogenesis of diabetic kidney disease (DKD), and that hyperglycemia also contributes to cellular senescence in DKD [55,56]. Although renal cellular senescence is the underlying cause of age-related parenchymal glomerular or tubular cell shedding or loss, cellular senescence does not always play an adverse role in all renal diseases. For instance, autosomal dominant polycystic kidney disease (ADPKD) is due to uncontrolled proliferation of renal tubular epithelial cells, and the CDK inhibitor, roscovitine, could mitigate the progression of ADPKD mainly by promoting cellular senescence [57,58]. Currently, although the characteristic alterations of aging kidneys have been well described, the distinction between normal aging and age-related kidney diseases merits further elucidation. Understanding the etiologies of renal aging and age-related renal diseases might enable the rational development of prophylactic interventions as well as novel targeted treatment strategies for renal dysfunction. The results of the current studies suggest that epigenetic changes induced by various etiologies (e.g., uremia, cellular senescence, psychological, lifestyle, various pathogens) may precipitate the development of age-related kidney diseases (Figure 2) [24]. Meanwhile, it is increasingly recognized that epigenetic modifications in embryonic and adult kidney development are interconnected and dysregulation influences age related kidney diseases [12]. Thus, a further understanding of epigenetic modification regulation in embryonic and adult kidney development will improve our understanding of molecular mechanisms of age-related kidney diseases and develop new treatments against these diseases.

Taken together, from the current studies, we can conclude that despite great progress being made in the understanding of the renal aging process and the associated decline in renal function, the pathogenesis of age-related kidney diseases still remains unanswered and warrants further investigation.

## 3. Epigenetics and Age-Related Kidney Diseases

Currently, the mechanisms responsible for age-related decline in organ function have not been fully understood, but growing evidence suggests that epigenetic alterations, primarily including aberrant DNA methylation, histone post-translational modifications, and regulation by ncRNAs, play an important role in various age-related human disorders such as neurodegenerative [59], cardiovascular diseases [60], and degenerative spinal stenosis [61] as well as various kidney diseases [24,62]. In the following sections, we will summarize the current knowledge on the implications of epigenetics in age-related kidney diseases.

### 3.1. DNA Methylation in Age-Related Renal Diseases

Although covalent modifications of DNA bases had been described since 1948 by Hotchkiss [63], it was not until 1969 that Griffith and Mahler proposed that these modifications may involve the modulation of gene expression [64]. In particular, the major modification in eukaryote DNA is 5-methylcytosine (5mC) [65], which primarily occurs at the fifth position of the pyrimidine ring of cytosines. DNA methylation, the first identified epigenetic mechanism, which occurs primarily at cytosine-phosphate-guanine (CpG) dinucleotides within the gene promoter regions [66], is involved in regulating gene expression through inhibiting specific transcription factors binding to DNA or recruiting mediators of chromatin remodeling (e.g., histone-modifying enzymes) [67,68,69,70]. In mammals, DNA methylation patterns are routinely established and maintained by three DNA methyltransferases (DNMT) [70,71] including DNMT1, DNMT3a, and DNMT3b, while in contrast, DNA demethylation can be achieved by the ten-eleven translocation (TET) enzymes through converting 5mC to 5-hydroxymethylcytosine (5hmC) [72,73].

The dynamic regulation of DNA methylation and demethylation is one of the most important epigenetic regulatory mechanisms in eukaryotic cells, which up to now, has not been fully understood. Increasing evidence has demonstrated that aberrant DNA methylation of specific CpG sites may serve as sensitive biomarkers to identify individuals at risk for age-related diseases [61,74]. In particular, age-related renal diseases such as CKD and ESRD are a major public health problem worldwide for its high morbidity among aging populations. Several studies have well explored the associations between DNA methylation patterns and age-related kidney diseases [75,76,77]. For instance, a recent study investigated the genome-wide changes in DNA methylation in renal biopsy samples obtained from 95 healthy kidney donors aged from 16 to 73 years old [11]. A total of 92,778 CpG methylation sites were identified to be significantly associated with donor age through the analysis of genome-wide changes in DNA methylation (more than 800,000 CpG sites) (FDR <0.05), corresponding to 10,285 differentially methylated regions. Interestingly, these regions were most frequently located in the Wnt/β-catenin signaling pathway related genes including the dickkopf Wnt signaling inhibitors (*DKK*), several *SOX* transcription factors, Wnt inhibitory factor 1 (*WIF1*), secreted frizzled related protein 2 (*SFRP2*), retinoic acid receptor alfa and beta (*RARA* and *RARB*), and so on. Hypermethylation in the promoter region of these Wnt signaling inhibitor genes may contribute to the activation of Wnt/β-catenin signaling in aged kidney. Furthermore, Wnt/β-catenin signaling, a conserved signaling pathway in organ development, is kept silent in normal adult kidneys [78,79], which is reactivated predominately in tubular epithelial cells in a variety of CKD models [80]. Thus, hypermethylation of these Wnt signaling inhibitor genes induce activation of Wnt/β-catenin signaling, which may lead to aging-related renal changes by triggering tubular epithelial cell transition to mesenchymal or senescent phenotype and promoting renal fibrosis. This study clearly revealed a causal relationship between DNA hypermethylation and age-associated renal dysfunction [11], indicating that DNA methylation alterations could be a new class of potential non-invasive diagnostic and prognostic biomarkers for age-related kidney diseases. Moreover, numerous clinical observations and animal studies have demonstrated that DNA methylome alterations are implicit in the development and progression of CKD [81,82,83,84]. For example, an epigenome-wide association study (EWAS) was performed to investigate the genome-wide methylation profiles in whole blood samples from 4859 aging adults, which demonstrated that the epigenetic signatures were significantly associated with kidney function and CKD as well as with the clinical endpoint renal fibrosis [84]. In this study, the researchers identified 19 CpG sites associated with eGFR and CKD from whole blood samples, among which five CpG sites were associated with renal fibrosis and showed consistent and significant DNA methylation changes in renal cortical biopsy samples from CKD patients. The study revealed that eGFR-associated CpG sites were significantly enriched in regions bound to serval transcription factors including Early B-cell Factor1 (EBF1), E1A Binding Protein P300 (EP300), and CCAAT/enhancer-binding protein beta (CEBPB), highlighting the impact of epigenetic modifications on renal function. Moreover, previous studies have demonstrated that several targeted genes regulated by CEBPB, EBF1, and EP300 are essential for kidney development and function [85,86,87,88], suggesting that methylation alterations of CEBPB, EBF1, and EP300 target genes may block the regulation of CEBPB, EBF1, and EP300 on their target genes, leading to the development of CKD. Thus, CEBPB, EBF1, and EP300 may serve as promising candidates for future experimental studies to illuminate the underlying gene regulatory mechanisms linking differential DNA methylation to kidney function in health and disease.

Despite several genome-wide association studies and epigenome-wide association studies having identified significant changes in DNA methylation with aging and age-related kidney diseases, there is currently still a lack of a direct evidence indicating that alterations in particular gene expression patterns as well as gene-specific DNA methylation influence renal aging [17,18]. A more recent study has provided strong evidence for uncovering important epigenetic features of kidney aging [17]. Recently, Gao and colleagues reported that chronic injection of D-galactose (D-gal)-induced aging or natural aging kidneys led to significant inhibition of *KLOTHO* and antiaging factor nuclear factor erythroid-derived 2-like 2 (*NRF2*) expression, accompanied by increased expression of DNMTs (subtypes of DNMT1, DNMT3a, and DNMT3b) as well as hypermethylation of *NRF2* and *KLOTHO* gene promoter [17]. Administration of DNA-demethylating agent, SGI-1027 and OLP, effectively reduced DNA methylation of the NRF2 and KLOTHO promoter and alleviated D-gal-induced aging-related structural and functional alteration changes in mouse kidney. Notably, the anti-renal aging effects of SGI-1027 in D-gal-induced aging mice were significantly abolished by silencing *KLOTHO* in vivo. From this study, we can conclude that dysregulation of DNMT1/3a/3b significantly contributes to the kidney aging process and epigenetic intervention with DNA-demethylating agents can mitigate renal aging alterations, suggesting that alteration of particular gene expression patterns and genomic DNA methylation can indeed influence the renal aging process. Thus, developing therapeutic strategies aimed at reversing age-associated adverse epigenetic changes will contribute to the development of novel therapeutic interventions that can delay or alleviate renal aging and age-associated kidney disorders.

From current research on DNA methylation in age-related kidney diseases (Table 1), we can conclude that DNA methylation might have exerted critical regulatory functions in both normal renal aging and age-related kidney diseases. Nevertheless, the present studies are far from sufficient to elucidate the molecular mechanisms underlying DNA methylation changes in age-related kidney diseases. Furthermore, most of these investigations lack in vivo experimental validation. Therefore, more systematic studies focused on DNA methylation alterations in age-related kidney diseases and clinical applications are required in the future. 

### 3.2. Altered Balance of Histone Modifications in Age-Related Renal Diseases

Genomic information of eukaryotic cells is mainly deposited in nuclear chromatin, which consists of DNA, RNA, histones, and non-histone proteins [89,90]. In particular, the chromatin displays two very different states: euchromatin and heterochromatin [91,92], in which the former is loosely packed and transcriptionally active while the latter is tightly packed and less transcriptionally active. The histone octamer is composed of two histone H2A–H2B heterodimers and one H3–H4 tetramer, which is encapsulated by approximately 147 bp of DNA to form the nucleosome [93]. Histones are composed of a central globular domain and flexible charged N-terminal tails. Because the NH2 terminus of histones extend from the core and contain specific amino acid residues, they are highly susceptible to multiple types of post-translational modifications such as acetylation, phosphorylation, ubiquitination, SUMOylation, and methylation, thereby affecting their function [91,94,95]. Notably, histone modifications can directly affect chromatin structure through preventing the binding of transcription factors to their specific binding sites or altering the interaction between histone tails and nucleosome DNA, which play critical roles in the regulation of gene expression [96,97]. For instance, a number of genome-wide studies have indicated that histone modifications in a specific genomic region can contribute to changes in chromatin structure, leading to either the activation or repression of specific gene expression [98,99,100].

Histone modification alterations have been reported to influence a wide variety of biological processes (e.g., cell growth, cell differentiation, threonine metabolism, and inflammation [101,102,103,104]) that positively or negatively affect the development of aging. Growing evidence supports the idea that histone modifications have greatly increased our knowledge regarding epigenetic modifications in the expression of many genes during the development of renal aging [105,106]. When it comes to age-related kidney diseases, the most prevalent reported and best characterized type of histone modifications are histone acetylation/deacetylation [107], which are dynamically regulated by two families of enzymes with opposing roles: histone acetyltransferases (HATs) and histone deacetylases (HDACs) [108]. Many acetylation signs on histones typically reduce with age, mainly including H3 acetylation on lysine 18, 27, and 56 as well as bulk H4 acetylation, which are considered to contribute to the aging process and the development of age-related diseases [109]. At present, mammalian HDACs are divided into four categories according to their homology with yeast [110] including class I (HDAC1, 2, 3, 8), class II a (HDAC4, 5, 7, and 9), class II b (HDAC6 and 10), class III (Sirtuins, Sirt1-7), class IV (HDAC11), of which class I, II and IV HDACs are dependent upon Zn^2+^ as a cofactor and sensitive to all HDAC inhibitors, while class III HDACs rely on NAD+ for their activities and are insensitive to classical HDAC inhibitors [111]. Generally, HDACs can deacetylate lysine residues on histone tails, which restore the positive charges of chromatin histones, leading to the condensation of chromatin and inhibition of gene transcription. Emerging studies have indicated the aberrant expression of HDACs is closely associated with renal aging and age-related kidney diseases [107,112]. Moreover, accumulating evidence also suggests that HDAC inhibitors can beneficially modulate age-related processes, probably by reversing age-related deacetylation of chromatin, acetylation of histones near pro-longevity genes, activating pro-longevity proteins, and/or de-activating anti-longevity proteins [109,113], which still needs to be further investigated.

Histone deacetylase 3 (HDAC3), a member of class I HDAC family, is critical for mammalian embryonic development and the aging process. The aberrant activation of HDAC3 is closely linked to a wide variety of human disease such as cancer [114], diabetes mellitus [115,116], neurodegenerative disorders [117], and CKD [25]. For instance, *NM_026333*, a newly discovered anti-aging gene, was identified to be obviously downregulated in the kidneys from coupling factor 6 (CF6)-overexpressing transgenic and high salt-fed mice present with premature aging-like phenotypes [118]. Endogenous overexpression of *NM_026333* or supplementation of NM_026333 recombinant protein alleviated CF6-induced senescence characteristics of HEK-293 cells (e.g., impaired autophagy, genomic instability, and epigenetic alterations) through abolishing HDAC3-induced transcriptional repression of autophagy related 7 (*Atg7*), suggesting that HDAC3 can accelerate renal aging by impairing autophagy [118]. Moreover, HDAC3 has been indicated to inhibit the transcription of *Klotho*, a well-known anti-aging protein, in various kidney diseases such as CKD [25] and renal fibrotic disorders [26]. Targeting Klotho loss through HDAC3 inhibition may serve as a new strategy for anti-renal fibrosis therapies as well as promising therapeutic potential for a reduction in CKD progression. These studies together indicate that aberrant activation of HDAC3 likely contributes to age-related kidney diseases, however, whether target inhibition of HDAC3 might mitigate renal aging as well as prevent and treat age-related kidney diseases remains to be further investigated.

Sirts, a conserved family composed of seven members (Sirt1-7), belong to class III NAD+-dependent HDACs, which have been considered as a class of critical regulators of aging and metabolic disease [119]. Sirts are widely expressed in various types of kidney cells, some of which have been demonstrated to delay renal aging. The expression of Sirt1 was significantly decreased in aging kidneys, which was associated with changes in the expression of a variety of target molecules (e.g., *Klotho* [120], peroxisome-proliferator-activated receptor-γ coactivator-1α (PGC-1α) [121], forkhead box O3 (*FOXO3*) [121,122], AMP-activated protein kinase (AMPK) [123], and hypoxia-inducible factor-1α (*HIF-1α*) [124]). For example, podocyte-specific silencing of *Sirt1* was found to exacerbate age-related glomerulosclerosis and albuminuria [121]. Endothelial *Sirt1* deficiency contributes to nephrosclerosis through the downregulation of matrix metalloproteinase-14 (*MMP-14*), which is primarily associated with vascular aging and fibrosis [125] while pharmacological activation of Sirt1 significantly decreased the pathologic changes of aging in the kidney via activation of AMPK and the PPARα signaling pathway [123]. Consequently, Sirt1 is considered as a potential therapeutic target to treat age-related kidney diseases. Sirt3, a critical regulator of cell senescence, is related to renin-angiotensin-aldosterone system (RAAS) activation, which has been reported to play an important role in renal aging. Previous study has shown that targeted knockout of the *Agtr1a* (*Agtr1a*−/−) gene encoding Ang II type 1 receptors (AT1R) significantly extend the life span of mice, which is attributed to the attenuation of oxidative stress and the upregulation of Nampt and Sirt3 [126]. However, one more recent study by Uneda et al. has reported conflicting results and indicates that it is Sirt1, but not Sirt3, that is significantly decreased in the kidneys of aged *Agtr1a*−/− mice [127]. Although the critical function of Sirt3 in the renal aging process through the Ang II-AT1R signaling pathway remains controversial, *Sirt3*−/− mice develop more serious renal fibrosis than their age matched wild-type (WT) littermate controls as they age [128]. Altogether, these results suggest that Sirt3 does indeed play an important role in ameliorating age-related kidney diseases possibly through the attenuation of oxidative stress and the maintenance of mitochondrial integrity. In addition, the participation of Sirt6 has also been indicated to ameliorate age-related kidney damage through the suppression of the pro-inflammatory nuclear factor kappa-B (NF-Κb) signaling pathway [129]. Sirt6 deficiency significantly results in the progression of glomerular injury in aged mice kidney [130]. Taken together, current findings suggest that Sirts ameliorates the degree of tissue damage and fibrosis in the aging kidneys, probably through decreasing oxidative stress and inflammation, indicating that Sirts has great potential as novel therapeutic targets for the prevention and clinical treatment of age-related kidney diseases. Thus, great efforts toward demonstrating that Sirt activators are of great beneficial to patients would yield major clinical and public health implications.

In general, the above research demonstrates the underlying roles of histone acetylation and deacetylation in modulating renal aging and age-related renal diseases (Table 2). However, these studies provided limited insight into delineating specific molecular mechanisms, and direct evidence from in vivo studies supporting abnormal histone modifications patterns that contribute to age-related renal disease is lacking. Thus, continued efforts are still needed to elucidate the role of histone modifications in the field of age-related renal disorders.

### 3.3. Non-Coding RNA in Age-Related Renal Diseases

About 93% of human genomic DNA can be transcribed into RNAs [131], of which only approximately 2% of these RNAs can be translated into proteins, and the remaining RNAs represent ncRNAs that do not have protein encoding ability or have very low protein coding capability [132,133]. In recent years, the critical roles of ncRNA transcripts in epigenetic gene regulation have been increasingly highlighted [134,135]. Accumulating evidence suggests that ncRNAs, mainly including microRNAs (miRNAs), long ncRNAs (lncRNAs), and circular RNAs (circRNAs), play important roles in senescence and aging [136,137,138], with several studies conducted in the context of renal aging, and have been regarded as potential biomarkers and therapeutic targets [139].

MiRNAs are a large class of short ncRNAs of ~22 nucleotides in length that are highly conserved across species, which can induce gene silencing through recruiting Argonaute (AGO) proteins to target sites predominantly within the 3′untranslated region (3′UTR) of mRNAs [140]. Mature miRNAs are bound by Ago proteins to form the miRNA-induced silencing complex (miRISC), which can induce the degradation of target mRNAs or translation inhibition [141]. Increasing evidence has indicated that dysregulated expression of miRNA is associated with various human diseases such as cancer [142], age-related kidney disorders [21], and so on. In recent years, accumulating epigenetics data have revealed the involvement of miRNAs in age-related kidney diseases. Bai and colleagues performed the first in-depth miRNA-expression profiling in kidney tissues from 3- and 24-month-old rats and identified significant age-related changes in miRNA expression in aged kidneys [21]. In this study, the researchers identified 18 miRNAs that were significantly upregulated and seven miRNAs that were remarkably downregulated with aging in rat kidneys. The upregulated miRNAs were mainly related to the regulation of genes involved in cell proliferation, antioxidative defense, and energy metabolism as well as extracellular matrix degradation, while the downregulated miRNAs predominantly targeted genes linked to cell-cycle arrest and immune inflammatory response. *miR-335* and *miR-34a* were significantly upregulated in the aged kidney tissues and aging mesangial cells, and could induce premature senescent phenotypes in young mesangial cell via modulating their target genes, superoxide dismutase 2 (*SOD2*) and thioredoxin reductase 2 (*Txnrd2*), through binding to the 3′UTR of each gene [21]. Consistently, a recent study indicates that aberrant upregulation of miR-34a contributes to renal fibrosis progression though direct binding with the 3′UTR of *Klotho*, a key gene controlling aging [143]. In addition, accumulating studies have reported the involvement of some other dysregulated miRNAs in renal aging and/or contribution to age-related kidney diseases, mainly including *miR-200c* [22], *miR-133b* [23], *miR-184* [144], *miR-150* [144], and *miR-21* [22,145,146]. Although there is little direct evidence that miRNAs play a role in age-related kidney diseases, a large body of evidence indicates that miRNAs are closely related to renal fibrosis and represent a potential target in the treatment of renal fibrosis [147] such as *miR-103a-3p* [148], *miR-146b-5p* [149], *miR-204* [150], *miR-29b* [151], *miR-150*, and *miR-495* [152]. Since renal aging is often accompanied by renal fibrosis, these renal fibrosis-related miRNAs may also play critical roles in renal aging as well as in age-related kidney diseases, which deserves to be further investigated. Collectively, the involvement of many different miRNAs in age-related kidney diseases indicates that a panel of miRNAs would potentially be more useful than a single miRNA as a biomarker. Further research is still needed in this field.

LncRNAs are another large class of ncRNAs derived from the genome regions, which are more than 200 nucleotides in length with no protein-coding potential [153,154]. Growing evidence has demonstrated that lncRNAs are involved in various biological processes including aging and age-related diseases [155,156] by regulating gene expression at the level of chromatin modification, transcription, and post-transcriptional processing [157]. Likewise, an increasing number of discoveries have highlighted the critical roles that lncRNAs play in age-related kidney diseases. One recent study reported that *NEAT1*, a novel lncRNA, could impair the protective effect of Klotho against renal tubulointerstitial fibrosis and epithelial–mesenchymal transition (EMT) in human proximal tubular cell line (HK-2) cells in DKD [158]. In this study, in order to screen for lncRNAs associated with Klotho expression, the authors detected EMT-related lncRNAs (*Gas5, H19, Hotair, Hottip, Malat1, Meg3, Miat, Neat1, Pvt1, and Tug1*) expression patterns in the kidney tissues from Klotho-overexpressing diabetic mice and found that *Neat1* expression was notably decreased. Further studies indicated that Klotho delayed the progression of renal tubulointerstitial fibrosis and renal tubular EMT in DKD through downregulating *NEAT1* expression. *LncRNA-ATB*, upregulated in TGF-β-treated HK-2 cells, was found to contribute to cell senescence induced by TGF-β via the TGF-β/Smad signaling pathway [159], but direct experimental evidence in vivo is lacking. More recently, Tamás et al. performed in-depth lncRNA profiling in the kidney tissues from adult mice (10 months) and aged mice (26–30 months) subjected to unilateral renal IRI to investigate the effects of aging and unilateral IRI on the expression of renal lncRNAs [28]. Researchers revealed that IRI had the greatest impact on kidneys in aged mice compared to adult mice, because eight lncRNAs were significantly upregulated by ischemia in age mice, whereas only four lncRNAs were remarkably upregulated by ischemia in adult mice. *IGF2AS* was upregulated by IRI only in the kidneys of old mice. Notably, inhibition of IGF2AS expression was found to be protective in different disease models including local-anesthetic induced neurotoxicity [160] and diabetes [161], suggesting that *IGF2AS* may play a detrimental role in ischemia-reperfusion injury in aged kidneys. *Y RNAs*, the only lncRNAs that were affected by aging, have been indicated to be involved in several age-related processes [162,163] such as cell proliferation, stress responses as well as inflammation, but their exact role in renal aging has not been illustrated. Ingenuity pathway analysis (IPA) indicated that H19 downregulation was linked to aging through the p53/TP53 pathway. Moreover, suppression of *H19* has been found to be associated with the increased expression of *p16* and *p21* in several studies [164,165]. Taken together, from the current studies, we can conclude that lncRNA could be considered as a potential therapeutic target for age-related kidney diseases, which needs further investigation. However, it is worth noting that, unlike miRNAs and circRNAs, lncRNAs are poorly conserved across species, so their targets and functions should be interpreted with caution.

CircRNAs are a novel class of endogenous expressed ncRNAs with a covalently closed structure and single-stranded that are derived from exons or introns, which can act as miRNA sponges, transcription regulators, or RNA-binding protein (RBP) partners to regulate target gene expression [166,167]. Additionally, compared with miRNAs and lncRNAs, circRNAs exhibit greater stability as well as higher conservation. Recent studies have demonstrated that circRNA expression levels change with age in various tissues of multiple species, ranging from nematodes to mammals [138]. For example, circFoxo3, derived from exon 2 of *FOXO3* gene, has been demonstrated to be remarkably upregulated in the heart tissues of aged mice and humans and is associated with extensive senescence [168]. Inhibition of endogenous circFoxo3 could attenuate senescence and alleviate cardiomyopathy. Further studies have indicated that *circFoxo3* can bind to senescence-related protein inhibitor of DNA binding 1 (ID1) and E2F transcription factor 1 (E2F1) as well as stress-related proteins HIF-1α and focal adhesion kinase (FAK), thereby blocking anti-senescent function of these proteins. In addition, a more recent study identified *circGRIA1* as a key moderator of synaptic plasticity and synaptogenesis in rhesus macaque brain [169]. *CircGRIA1* expression is remarkably increased in the aging macaque brain, which could directly bind to its host gene promoter and suppress the expression of its parental gene, thus contributing to brain aging. At present, although a large number of studies have confirmed that circRNAs play crucial roles in the development and progression of aging and age-related diseases in many organs [138], the exact roles of circRNAs in renal aging as well as age-related kidney diseases remain elusive. Unlike miRNAs and lncRNAs that have been well studied in aged kidneys, critical age-related circRNAs still remain to be profiled, in detail, in future studies. Since circRNAs are far more stable than other ncRNAs, they can stably exist in various body fluids, making them one of the most desirable biomarkers for the diagnosis and treatment of various aging related diseases including age-related kidney diseases.

Collectively, the above studies indicate that ncRNAs including miRNAs, lncRNAs, and circRNAs may play critical regulatory roles in the processes of renal aging as well as age-related kidney diseases (Table 3), which may serve as promising diagnostic biomarkers and therapeutic targets clinically. However, the functional roles of most ncRNAs, especially circRNAs, in renal aging fields are yet to be revealed. Therefore, it calls for more comprehensive research in this field to demonstrate and validate the molecular mechanisms in depth.

## 4. Epigenetics Provides Potential Diagnostic and Therapeutic Targets for Age-Related Kidney Diseases

In contrast to genetic changes, epigenetic alterations are potentially plastic and reversible, which can be therapeutically manipulated for the treatment of indicated diseases [170,171]. Many epigenetic hallmarks, mainly including DNA methylation, histone modifications, and ncRNAs, have displayed great potential in diagnosis, prognosis, and monitoring treatment as well as predicting therapy response in a variety of neoplastic and non-neoplastic diseases [172,173,174,175]. For instance, methylation levels of promoter regions of cyclin-dependent kinase inhibitor 2A (*CDKN2A*), cadherin 13 (*CDH13*), ras association domain family member 1 (*RASSF1A*), and adenomatous polyposis coli (*APC*) are associated with the early recurrence of stage I non-small cell lung cancer (NSCLC) after curative surgery [176]. A phase III trial confirmed that tucidinostat, an oral subtype-selective HDAC inhibitor, has been proven to be effective in patients with hormone receptor-positive (HR+) breast cancer [177]. Besides, some HDAC inhibitors and DNMT inhibitors have also been approved for the clinical treatment of several non-oncological diseases such as lupus [178], heart failure [179], and schizophrenia [180]. In addition, as miRNAs are the most extensively researched ncRNAs, certain miRNAs such as *miR-16* [181] and *miR-34* [182,183] have already entered preclinical and clinical trials for the treatment of human diseases. Given the critical role of epigenetics in modulating renal aging and age-related kidney diseases, it is promising that these epigenetic alterations could also be used to develop specific biomarkers and therapeutic targets to combat these age-related diseases.

**Figure 3:**
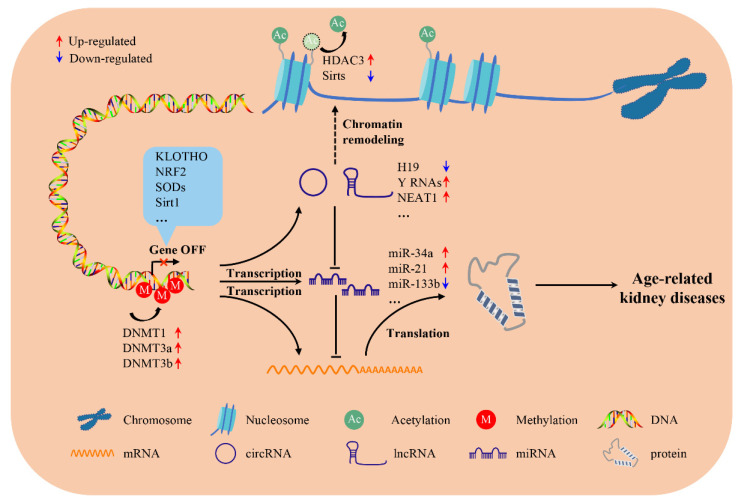
Schematic diagram of the mechanisms of the main epigenetic alterations in age-related kidney diseases. Aberrant DNA methylation, abnormal histone modifications as well as regulation by ncRNAs are associated with age-related kidney diseases. These changes have been implicated in age-related kidney diseases through the alteration of certain genes expression in various kidney cells.

Notably, as described above, aberrant DNA methylation, abnormal histone post-translational modifications as well as regulation by certain ncRNAs have contributed to age-related kidney disorders (Figure 3). Hence, it is tempting to speculate that multiple different manipulations to treat and prevent age-related kidney diseases such as the development of novel DNA-demethylating agents, HDAC inhibitors, and specific ncRNA blockers and agonists. Nevertheless, so far, none of the current available epigenetic modifiers have been authorized to enter preclinical or clinical trials in the field of renal aging. Thus, in the near future, there is an urgent need for more experimental studies to further exploit the promising application of epigenetic agents in battling against age-related kidney diseases.

## 5. Summary and Future Prospects

Here, we attempted to provide an overview of the main epigenetic modifications involved in the renal aging process and their role in age-related kidney diseases. Currently, available data indicate that the main types of epigenetic modifications including DNA methylation, histone modifications, and ncRNAs modulation have been implicated in the progression of renal aging and age-related kidney diseases. Nonetheless, prospective clinical studies of large cohorts are still needed to further determine the feasibility of using these epigenetic molecular markers for the prevention, diagnosis, and treatment of age-related kidney diseases before going into future clinical applications.

Our current understanding of epigenetic regulation in age-related kidney diseases is fragmentary and still in a relatively preliminary stage. Based on the results of this review, we proposed potential areas for future research that may yield novel insights into the role of epigenetics in age-related kidney diseases. As the epigenome undergoes massive alterations during the aging processes, it still remains a challenging issue to discriminate the causes from consequences as well as to determine the degree to which epigenetic alterations drive the aging process [184]. Moreover, it remains unclear how age-related epigenetic changes directly contribute to the development of various age-related kidney diseases. Thus, elucidating the causal relationship of epigenetic changes to the physiological process of renal aging is of great significance in future research, which will deepen the understanding of the epigenetic mechanisms of aging, provide novel clues for delaying renal aging, and prevent aging-related renal diseases. Another potential future direction should be to conduct in-depth study to reveal the roles of circRNAs in the field of renal aging and age-related renal diseases. Due to the unique features and remarkable stability of circRNAs, age-dependently altered circRNAs have been identified as promising biomarkers of aging and age-related diseases [138]. Moreover, the levels of many circRNAs have been shown to change with aging in a tissue-specific manner, which provides evidence of their function in the aging of specific tissues. However, circRNAs are still in their infancy in the field of renal aging research. Therefore, further elucidation of the specific roles and mechanisms of circRNAs in the development of renal aging are of great value for the diagnosis, treatment, and prevention of age-related kidney disease as well as in enriching our understanding of epigenetic regulatory mechanisms underlying age-related kidney diseases.

Currently, many epigenetic regulators have been identified involved in the aging process of the kidney, which are potential targets for improving the aging phenotype in kidney such as DNMT1/3a/3b, HDAC3, Sirt1, *miR-335*, *miR-34a*, and so on. The development of novel agents targeting these epigenetic regulators may yield novel therapeutic strategies for the prevention and/or treatment of age-related kidney diseases, and their potential utility in animal models and humans merits further investigation.

This work was supported by the National Natural Science Foundation of China grants No. 81974162, No. 81671066 (to prof. X.M.).

## Figures and Tables

**Table 1:** DNA methylation in age-related kidney diseases.

Allele	Sample	Dysregulation	Target	Brief Role	Reference
DNMT1 DNMT3a DNMT3b	Mice (2–25 months)	Upregulated	*KLOTHO*, *NRF2*	Aberrant DNMT1/3a/3b elevations resulting in the promoter hypermethylation and expression suppressions of *KLOTHO* and *NRF2*, which accelerate renal fibrosis in D-gal-induced aging.	[17]
DNMTs	Mice (12–14 and 75–78 weeks)	Upregulated	*SODs*, *Sirt1*, *Nox4*	Hypermethylation of antioxidant enzymes induced by DNMTs in the aged kidney during hypertension worsens redox imbalance leading to kidney damage.	[18]

**Table 2:** Histone modifications in age-related kidney diseases.

Allele	Sample	Dysregulation	Target	Brief Role	Reference
HDAC3	HDAC3 knockout mice	Upregulated	*Klotho*	Aberrant HDAC3 induction contributes to renal fibrogenesis through inhibiting Klotho, a renal epithelium-enriched aging suppressor.	[26]
Sirt1	Mice (5, and 26–28 months)	Downregulated	*PGC1α*, *FOXO3/4*, *NF-κB*	Podocyte-specific Sirt1 knockdown accelerates kidney injury in aging mice through Sirt1-mediated deacetylation of downstream targets.	[121]
Sirt1	Mice (3 and 24 months)	Downregulated	*FOXO3*	Long-term calorie restriction-induced upregulation of Sirt1 in aged kidney attenuated hypoxia-associated mitochondrial and renal damage by enhancing Bnip3-dependent autophagy.	[122]
Sirt1	Mice (24 months)	Downregulated	AMPK and PPARα signaling	Pharmacological activation of Sirt1 by resveratrol attenuates age-related renal injury through activation of AMPK and PPARα signaling.	[123]
Sirt1	Mice (5 weeks and 24 months)	Downregulated	*HIF-1α*	Sirt1-induced deacetylation of *HIF-1α* may have protective effects against tubulointerstitial damage in aged kidney.	[124]
Sirt1	Mice (12 weeks and 15 months)	Not mentioned	*MMP-14*	Endothelial Sirt1 deficiency contributes to nephrosclerosis through downregulation of *MMP-14*, which is primarily associated with vascular aging and fibrosis.	[125]
Sirt1	Mice (3–4, 11–12, and 22–25 months)	Down-regulated	Not mentioned	Angiotensin II type 1 receptor-associated protein (ATRAP) plays an important role in inhibiting kidney aging, possibly through sirt1-mediated inhibition of oxidative stress.	[127]
Sirt3	Mice (15 months WT and Sirt3-KO)	Downregulated	Glycogen synthase kinase-3 beta (GSK3β)	Sirt3 blocks aging-associated renal fibrosis through deacetylating and activating GSK3β.	[128]
Sirt6	Mice (6 and 24 months)	Downregulated	NF-kB signaling	CR-induced Sirt6 activation delays age-dependent renal degeneration through suppressing the NF-kB signaling pathway.	[129]
Sirt6	Mice (1, 2, 7 months WT and Sirt6-KO)	Downregulated	Unknown	Sirt6 deficiency results in progression of glomerular injury in the aged mice kidney	[130]

**Table 3:** Non-coding RNAs in age-related kidney diseases.

ncRNAs	Sample	Dysregulation	Target	Brief Role	Reference
*miR-335* *miR-34a*	Rat (3 and 24 months)	Upregulated	*SOD2* *Txnrd2*	*miR-335* and *miR-34a* may promote renal aging by inhibiting mitochondrial antioxidative enzymes *SOD2* and *Txnrd2*, respectively.	[21]
*miR-21* *miR-200c*	Mice (4–6, 18, 26–32 months)	Upregulated	*Sprouty-1* *ZEB2*	*miR-21* and *miR-200c* may promote aging-related renal fibrosis through inhibiting the growth factor signaling inhibitor *Sprouty-1* and inhibiting *ZEB2* translation, respectively.	[22]
*miR-133b*	Mice (24-Month control and UUO-treated)	Downregulated	*Connective tissue growth factor (CTGF)*	Overexpression of *miR-133b* inhibits renal fibrosis in aged mice with UUO through downregulating the expression of *CTGF*.	[23]
*miR-184* *miR-150*	Rat (3 and 24 months)	Upregulated	*Rab1a/Rab31*	*miR-184* and *miR-150* promotes glomerular mesangial cells (GMCs) aging through impairing autophagy and increasing oxidative injury.	[144]
*miR-21*	Rats (16 month and 3 month)	Upregulated	PPARα-HIF-1α signaling	Downregulation of *miR-21* could alleviate age-related fibrosis of kidney.	[145]
*miR-21*	Mice (12–14 and 75–78 weeks)	Upregulated	*CBS*, *CSE*, *MMP9*, *col IV*	Inhibition of *miR-21* rescued the old kidney dysfunction due to IRI by increasing H2S levels, reduction of macrophage-mediated injury, and promoting reparative process.	[146]
*Y RNAs* *Six3os*	IRI mice (10 and 26–30 months)	Upregulated	Unknown	May play a deleterious role in the development and progression of IRI-induced renal fibrosis in aged kidneys.	[28]
*H19*	IRI mice (10 and 26–30 months)	Downregulated	p53/TP53 signaling	IPA indicated that *H19* downregulation was linked to aging through p53/TP53.	[28]
*NEAT1*	Diabetic mice	Upregulated	ERK1/2 signaling	Knockdown of *NEAT1* can reverse renal tubulointerstitial fibrosis and renal tubular EMT caused by Klotho silencing.	[158]
*LncRNA-ATB*	HK-2 cells	Upregulated	TGF-β/Smad2/3 signaling	Silencing *lncRNA-ATB* attenuates HK-2 cells senescence induced by TGF-β	[159]

## Data Availability

All figure and tables are included within the manuscript.
